# Development of a Multiplex Real-Time PCR Assay for Predicting Macrolide and Tetracycline Resistance Associated with Bacterial Pathogens of Bovine Respiratory Disease

**DOI:** 10.3390/pathogens10010064

**Published:** 2021-01-13

**Authors:** Enakshy Dutta, John Dustin Loy, Caitlyn A. Deal, Emily L. Wynn, Michael L. Clawson, Jennifer Clarke, Bing Wang

**Affiliations:** 1 Department of Statistics, University of Nebraska-Lincoln, Lincoln, NE 68583, USA; enakshy.dutta@huskers.unl.edu (E.D.); jclarke3@unl.edu (J.C.); 2 School of Veterinary Medicine and Biomedical Sciences, University of Nebraska-Lincoln, Lincoln, NE 68583, USA; jdloy@unl.edu (J.D.L.); caitlyn.deal@huskers.unl.edu (C.A.D.); 3 U.S. Meat Animal Research Center, Agricultural Research Service, United States Department of Agriculture, Clay Center, NE 68933, USA; emily.wynn@usda.gov (E.L.W.); mike.clawson@usda.gov (M.L.C.); 4 Department of Food Science and Technology, University of Nebraska-Lincoln, Lincoln, NE 68588, USA

**Keywords:** bovine clinical samples, prudent antibiotic use, culture independent, rapid detection, receiver operating characteristic, quantitative PCR

## Abstract

Antimicrobial resistance (AMR) in bovine respiratory disease (BRD) is an emerging concern that may threaten both animal and public health. Rapid and accurate detection of AMR is essential for prudent drug therapy selection during BRD outbreaks. This study aimed to develop a multiplex quantitative real-time polymerase chain reaction assay (qPCR) to provide culture-independent information regarding the phenotypic AMR status of BRD cases and an alternative to the gold-standard, culture-dependent test. Bovine clinical samples (297 lung and 111 nasal) collected in Nebraska were subjected to qPCR quantification of macrolide (MAC) and tetracycline (TET) resistance genes and gold-standard determinations of AMR of BRD pathogens. Receiver operating characteristic curve analysis was used to classify AMR based on the qPCR results. For lung tissues, the qPCR method showed good agreement with the gold-standard test for both MACs and TETs, with a sensitivity of 67–81% and a specificity higher than 80%. For nasal swabs, qPCR results passed validation criteria only for TET resistance detection, with a sensitivity of 88%, a specificity of 80% and moderate agreement. The culture-independent assay developed here provides the potential for more rapid AMR characterization of BRD cases directly from clinical samples at equivalent accuracy and higher time efficiency compared with the gold-standard, culture-based test.

## 1. Introduction

Bovine respiratory disease (BRD) is one of the most common and costly cattle diseases and affects 97% of feedlots, or 16% of cattle, with economic losses estimated to exceed 1 billion USD/year [1,2]. BRD is a disease complex with multiple contributing factors, including environment, viruses, bacteria, and the host [3]. Disease onset is commonly initiated by viral infections, which may suppress host defense mechanisms, allowing opportunistic bacterial pathogens to replicate and colonize deeper in the lung. Environmental factors such as crowding, poor ventilation, weather, and weaning increase stress and also reduce host immunity [4]. The coexistence of multiple viral and bacterial pathogens often increases the severity of BRD outbreaks, leading to higher mortality and morbidity rates and associated economic burdens [5,6].

Among pathogenic bacteria causing BRD in feedlot cattle and neonatal calves, *Mannheimia haemolytica* is the most frequently isolated, followed by *Histophilus somni* and *Pasteurella multocida* [1]. The multifactorial nature of BRD is often a challenge for disease management. Administration of antibiotic drugs for treatment and/or metaphylaxis (control and prevention) purposes may potentially select for resistance among bacterial pathogen populations [7]. Recent studies have shown that the frequency of antimicrobial resistance (AMR) in BRD bacterial pathogens in feedlots is increasing, particularly resistance to macrolides (MACs) and tetracyclines (TETs) [8], which are among the most commonly used antibiotics on beef cattle feedlots in the United States [9,10,11,12]. The development of AMR could compromise the effectiveness of antibiotics for the treatment and control of BRD, potentially leading to higher mortality and lower productivity among cattle populations. Increasing AMR in BRD pathogens may also threaten public health, as the resistance genes found in BRD pathogens are contained within integrative conjugative elements (ICEs) that could potentially be transferred from BRD pathogens to zoonotic bacteria [10]. Hence, tools for rapidly and accurately detecting potential AMR are critical components of outbreak monitoring by veterinarians and producers. Such tools could be used as adjuncts to existing culture-independent BRD pathogen detection methods that use similar technology and workflows [13]. In addition, the use of these tools would enable rapid evaluation of the potential efficacy of antimicrobial interventions and ultimately benefit the beef industry and support antimicrobial stewardship by enabling informed selection of optimal drugs for BRD therapies. 

Classic culture-based methods for determining AMR commonly involve isolating and identifying organisms on solid media and assessing the growth inhibition of isolated bacterial strains under a series of concentrations of the target antibiotic to determine the minimum inhibitory concentration (MIC) [14]. As described by the Clinical and Laboratory Standards Institute (CLSI), isolates are classified as susceptible (S), intermediate (I), or resistant (R) to an antibiotic based on the relationships between MIC measurements and “breakpoints”, which are usually determined by taking into consideration the clinical outcomes of infections when the antibiotic is used [15]. The culture-based approach is widely used and is still considered the “gold-standard” test by national and international surveillance programs for monitoring AMR [14]. However, this method requires the growth, isolation, and identification of the target pathogens in pure culture, which can be time consuming and challenging due to sample contamination or overgrowth with environmental organisms [13].

In recent decades, molecular methods for detecting and quantifying AMR genes have shown promise as potential alternatives to the gold-standard AMR detection [16]. Quantitative real-time polymerase chain reaction (qPCR), which uses hydrolysis probes to generate a fluorescence signal, enables real-time assessment of DNA amplification and quantification of the genetic materials in the original sample. The qPCR approach also permits multiplexed detection of several gene targets in a single reaction. A recent study demonstrated that this approach is extremely useful for detecting opportunistic bacterial pathogens in bovine clinical samples and has advantages over culture-based approaches, especially when multiple pathogens coexist in the presence of normal flora [13]. These advantages make qPCR a powerful tool for the detection of genes that confer AMR using existing sample types and workflows. The detection of AMR genes in clinical samples (lungs and respiratory swabs) would provide veterinarians and clinicians with information on the presence or absence of AMR genes within hours to guide therapy selection. Although the detection of AMR genes in a sample does not necessarily indicate that the resistance genes are carried by BRD pathogens, such detection can be used as an indicator for the potential risks of antimicrobial-resistant infections in BRD outbreaks. Although the use of qPCR for AMR detection in tandem with culture-independent pathogen detection assays has not yet been evaluated, such a combination would provide information on the co-presence of BRD pathogens and BRD-associated resistance genes [13]. 

Unlike the gold-standard method, which generates a phenotypic classification of AMR, qPCR assays generate cycle threshold (Ct) values, which are continuous values representing the number of cycles at which the fluorescence signal exceeds the threshold value (i.e., the background signal level) and are used to estimate the number of gene copies in the original sample. To accurately predict the AMR classification index using a molecular testing method, an extensive epidemiological evaluation of the method based on a comparison with gold-standard methods must be conducted. Hence, the objectives of the present study were to (1) develop a qPCR method for the quantification of MAC and TET resistance genes in BRD clinical samples; (2) determine the optimal cutoff to support translation of qPCR results to a phenotypic classification of AMR; and (3) evaluate the validity of AMR phenotypic classification by the qPCR assay.

## 2. Results

### 2.1. Characteristics of the Multiplex qPCR Assay

In silico analyses showed 100% identity of the primer and probe loci to strains of *M. haemolytica* and *P. multocida* (Table S1). For the *H. somni* strains, 100% identity to ICE*tetR* gene targets was observed for sequences in GenBank; the remaining three targets were not found in *H. somni* strains available at the time of analysis. These resistance genes are not unique to BRD pathogens and are found on the chromosomes and/or plasmids of non-BRD-causing microbes (Table S2). The sequence identity of the primers and probes with non-BRD pathogen bacterial species does not indicate off-target binding to other genes, as it is expected that these AMR gene sequences are found in various other bacterial species. 

Preliminary evaluation of the assay using the reference strains indicated that the multiplex qPCR assay detected all of the targets in the *M. haemolytica* reference strain and had sensitivity for all four targets of <3.2 CFU/mL, with Ct values ranging from 37.34 (ICE*tetR*) to 35.70 (*msrE*) at the lowest detectable concentration (Table 1). The assay also correctly classified all *M. haemolytica* reference strains that had been determined to contain the target genes (Table S3). Due to the lack of sequences for non-*M. haemolytica* BRD pathogens in GenBank or other databases, a panel of *H. somni* (35 strains) and *P. multocida* (5 strains) from BRD cases that had been subjected to MIC testing was also evaluated (Table S4). The detection of MAC resistance genes by the assay corresponded with phenotypic MAC and TET resistance in 26 and 28 of 35 *H. somni* strains, respectively. For *P. multocida*, genotypic results that agreed with the phenotypes for both TET and MAC resistance were obtained for all five strains. To validate assay specificity, we also tested ATCC reference strains of *Actinobacillus pleuropneumoniae*, *Mannheimia granulomatis*, *H. somni*, and *B. trehalosi* that do not exhibit phenotypic resistance. No qPCR targets were detected in any of these reference strains. The susceptibility of two bovine respiratory strains of *B. trehalosi* was also evaluated; as expected, the tetracycline-resistant (TET^r^) and macrolide-resistant (MAC^r^) strain was positive for all four targets, whereas the susceptible strain was negative for all targets.

### 2.2. Phenotypic Antimicrobial Resistance Based on the Gold-Standard Test

**Figure 1:**
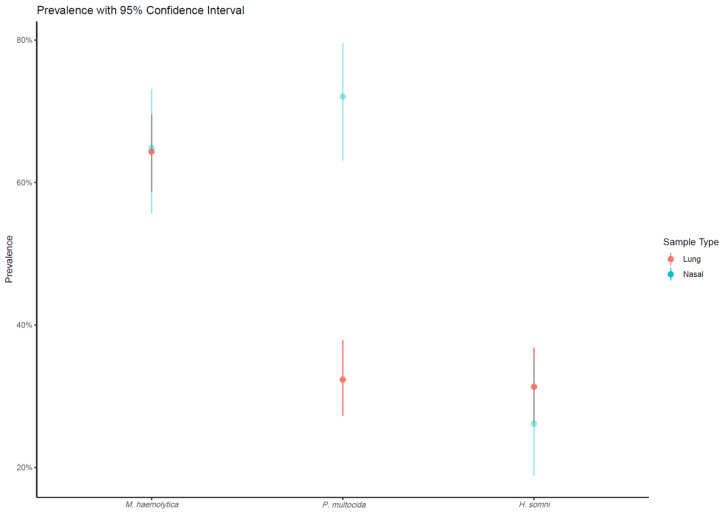
Prevalence of major bovine respiratory disease pathogens tested in this study by sample type.

**BRD pathogen detection.** Along with the sample distribution, Table 2 shows the prevalence of BRD pathogens by sample type. In addition to the individual BRD pathogens examined in this study, a new pathogen category, “positive for at least one BRD pathogen”, was evaluated. Overall, of the submitted samples for BRD diagnosis, at least one BRD pathogen was detected in 97.8%. In general, the prevalence of samples with at least one BRD pathogen was higher for lung samples (99.7%) than nasal samples (97.3%). Consistent with previous reports, *M. haemolytica* was the most frequently isolated BRD pathogen, comprising 63.7% of tested samples, followed by *P. multocida* (42.5%) and *H. somni* (29.6%). However, the pathogen distribution varied by sample type (Figure 1). Specifically, among lung samples, *M. haemolytica* was the most prevalent pathogen (64.3%), followed by *P. multocida* and *H. somni* at similar levels (32.3% and 31.3%). By contrast, both *M. haemolytica* and *P. multocida* were relatively highly prevalent in nasal samples (64.9% and 72.1%), whereas *H. somni* was not (26.1%).

**Phenotypic AMR data manipulation.** The distributions of AMR classifications determined by the MIC test by BRD pathogen and sample type are presented in Table 3. BRD pathogens isolated from clinical samples were classified as S, I, or R based on the comparison of the MIC test results with the breakpoints listed in Table 4. Strains classified as intermediate were much less prevalent, resulting in an inadequate number of samples in this category for statistical analysis. This was the major reason for combining R and I into the newly defined category R+I, resulting in a binary classification of AMR status based on the gold-standard MIC test, i.e., R+I and S. AMR status was recorded for individual pathogens as well as for the category of “positive for at least one pathogen”. In addition, resistance to two drugs in the MAC class was evaluated, and “resistance” to tilmicosin, tulathromycin, or MACs as a class (either tilmicosin or tulathromycin) was recorded.

**Resistant BRD pathogen isolation.** Based on the newly defined category R+I, the prevalence of TET^r^ BRD pathogens was higher than that of MAC^r^ pathogens for all pathogens and sample types. TET resistance prevalence ranged from 19.2% to 52.7%, while MAC resistance prevalence was between 8.8% and 39.3%. The only exception was *M. haemolytica* isolated from nasal samples, in which the prevalence of TET resistance (6.9%) was slightly lower than that of MAC resistance (8.3%). In addition, the occurrence of antimicrobial-resistant BRD pathogens was always higher in lung samples than in nasal samples. Specifically, 44.6% of lung samples and 23.1% of nasal samples possessed at least one TET^r^ pathogen, while 36.9% of lung samples and 19.4% of nasal samples possessed at least one MAC^r^ pathogen. The same trends were observed for all resistant BRD pathogens. 

### 2.3. Optimal Ct Cutoff Value Determination

**Figure 2:**
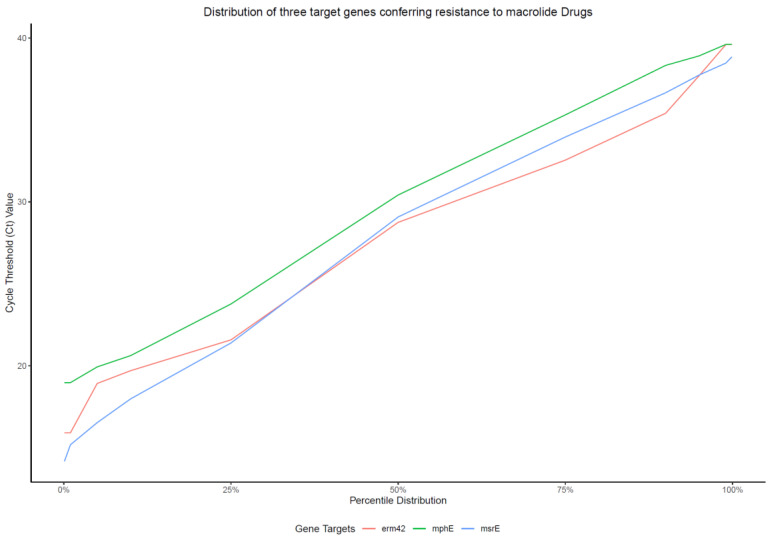
Consistent distributions of cycle threshold values quantified by the multiplex qPCR assay among three genes conferring resistance to macrolides.

**Real-time PCR data manipulation.** Three gene targets conferring resistance to MACs, i.e., *msrE*, *mphE*, and *erm42*, were detected using the qPCR method, and the average observed Ct value for the three targets was used. The use of the average value was deemed reasonable because of the high similarity of the distributions of the Ct values for the three genes (Figure 2). Although the distribution of the Ct values for *mphE* was slightly shifted from those for *erm42* and *msrE*, the percentile distributions were similar, so the average value for the three genes is a good representation of the inherent distribution of any of the three genes. Hence, all qPCR results for MAC drugs reported in this study are based on the average measurement of the three gene targets.

**Figure 3:**
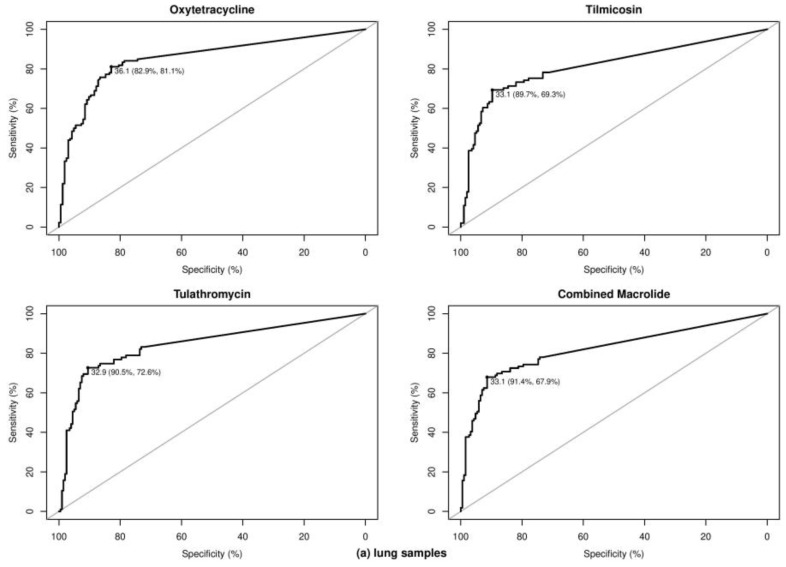

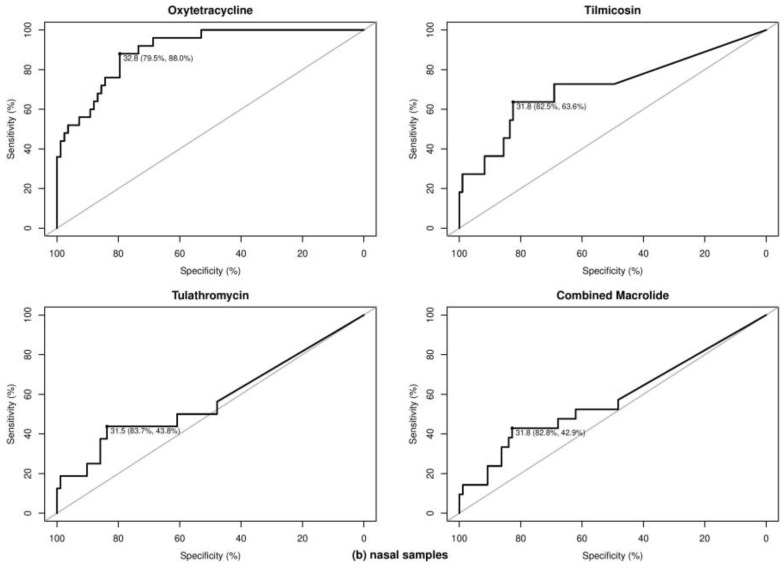
Receiver operating characteristic curves for determining optimal cycle threshold (Ct) cutoff values for lung samples (**a**) and nasal samples (**b**). Determined optimal Ct cutoff values with corresponding sensitivity and specificity were provided for predicting the presence of bovine respiratory disease pathogens resistant to tetracycline and macrolide drugs, respectively. Combined macrolide refers to resistance to either tilmicosin or tulathromycin.

**Optimal Ct cutoff value determination.** As shown in Table 5, for the lung samples, the optimal Ct cutoff value for TET was 36.06, with more than 80% sensitivity (Se) and specificity (Sp) and a good level of agreement with the MIC test (κ = 0.64). The prevalence of lung samples possessing at least one BRD pathogen resistant to TET was approximately 45%, leading to positive predictive value (PPV) and negative predictive value (NPV) estimates of 0.79 and 0.84; these values indicate that 79% of tested samples classified as resistant actually possessed at least one BRD pathogen resistant to TET and 84% of samples classified as susceptible were truly susceptible. Compared with TET resistance, the optimal Ct cutoff values for MAC resistance were lower, with lower Se but higher Sp of approximately 90%. The qPCR assay showed a good level of agreement with phenotypic MAC resistance, with κ ranging from 0.61 to 0.64. Similar to TET^r^ strain detection, both the NPV and PPV of the qPCR assay for MAC resistance were approximately 80%, ensuring relatively low false-negative and false-positive rates. Figure 3 shows the receiver operating characteristic (ROC) plots for all of the antimicrobials for the lung and nasal samples, respectively. In general, the performance of the qPCR approach for detecting resistance was lower for nasal samples than for lung samples. Se and Sp were both lower for nasal samples, with ranges of 50%–88% and 79%–83%, respectively; and the concordance with the MIC tests was fair to moderate, as indicated by κ values ranging from 0.30 to 0.58.

### 2.4. Validation of the Computational Approach 

**Determination of the required sample size for calculating the optimal cutoff.** The optimal Ct cutoff value was considered valid if the total sample size and the number of samples positive for resistant BRD pathogens were both greater than or equal to the minimum requirements established by considering the importance of both PPV and NPV. Estimates of the sample size necessary for the validity evaluation are listed Table S5. Clearly, the optimal Ct obtained for the lung samples for both TET and MACs satisfied the minimum requirement. For the nasal samples, only the optimal Ct values obtained for oxytetracycline and tilmicosin satisfied the requirements for both sample size and number of positive samples, whereas the optimal Ct values for tulathromycin and the combined MAC group did not satisfy the second requirement, i.e., the number of resistant samples included for determining the optimal Ct value was insufficient. If only NPV is of importance, then the optimal Ct obtained for all nasal samples is valid. However, increasing PPV or minimizing the false-positive rate helps reduce the overall misclassification rate and thus cannot be ignored completely.

**Cross-validation.** To validate the consistency of the optimal Ct cutoff value estimation, 5-fold and 10-fold cross-validation (CV) were conducted with a focus on the category of “resistance to at least one BRD pathogen”. Because more consistent estimates in terms of the optimal Ct cutoff and the κ value were obtained, 5-fold CV was used to optimize the optimal Ct cutoff value for the qPCR method. Table S6 shows the results of the 5-fold CV along with a comparison of the average level of agreement between the training and test sets. 

The optimal Ct values for the lung samples for both TETs and MACs were consistent in terms of required diagnostic accuracy. Overall, for the lung samples, the optimal Ct obtained from the overall data fell within the 95% average optimal Ct obtained using 5-fold CV. In addition, the average κ evaluated based on the training and test sets indicated a good level of agreement with the results of the MIC tests for both groups of antimicrobials. However, the optimal Ct obtained from the nasal samples was sufficiently validated only for the TET group. For the MAC group, a disparity in the average κ was observed between the training and test datasets. 

Table 6 summarizes the optimal Ct values and the level of agreement based on the minimum requirements for total sample size and resistant sample size and 5-fold CV. The optimal Ct for predicting the phenotypic AMR classification based on qPCR results was sufficiently validated for the lung samples containing MAC^r^ and/or TET^r^ BRD pathogens and for the nasal samples containing TET^r^ BRD pathogens. The optimal Ct values for the lung samples were 35.66 and 33.12 for TET and MAC resistance, respectively, with a good level of agreement between the gold-standard and the qPCR approach. The optimal Ct value obtained for the nasal samples was 33.27 for TET resistance, with a moderate level of agreement between the gold-standard and the qPCR approach.

## 3. Discussion

The method described in this study enables AMR gene detection in clinical samples in a culture-independent manner and thus can rapidly provide clinicians, veterinarians, and cattle producers preliminary information on the presence of potential AMR in BRD cases. Previous examinations of AMR in BRD pathogens have relied on whole-genome sequencing (WGS) approaches to mine genomic sequences for resistance genes. Although this approach is much more comprehensive, it is less practical for use with clinical samples and in situations where a clinician may need rapid results for drug decisions. Compared with isolates, working with clinical samples from livestock environments poses a significant challenge due to the large variations in sample types and quality, environment, and collection requirements. Working with clinical samples requires a targeted and quantitative approach such as qPCR, which has been shown to increase the detection of pathogens compared with culture-dependent methods such as WGS [13]. 

In developing this assay for use in a rapid format with clinical samples, our goal was to select robust gene targets with spatial and temporal consistency in BRD pathogen strain genomes and that confer resistance to highly relevant drug classes. Consequently, the performance of the method for both TETs and MACs was evaluated using ATCC- and clinical sample-sourced strains that have been subjected to MIC testing and/or WGS. Overall, the developed multiplex qPCR method performed well with high specificity for detecting the target AMR genes. In the initial validation, evaluation of the phenotypic–genotypic agreement using less comprehensive methods showed high levels of agreement for *M. haemolytica*, *P. multocida* and *B. trehalosi.* By contrast, for *H. somni*, disagreement between the results of the multiplex qPCR assay and phenotypic AMR classification using MIC was observed for 9 of 35 strains tested. Potential explanations for this discrepancy include alternative mechanisms of phenotypic resistance or inactive resistance genes in these strains. 

The results indicate that this method may be particularly useful for assessing the presence of TET or MAC resistance in BRD pathogens in general or in *M. haemolytica,* one of the most dominant bacterial pathogens. TETs and MACs were chosen as the target drug classes because they are often used as first-line treatments for BRD. More than 70% of drugs used for the treatment or prevention of BRD in the United States are MACs (including tulathromycin, tilmicosin, tildipirosin, and gamithromycin), followed by TETs (mainly oxytetracycline, ~9%) [9,17,18]. Determinants conferring resistance to MACs or TETs in the population of BRD pathogens are frequently carried on ICE elements, revealing a potential challenge in the use of these important drugs for BRD disease treatment and/or prevention [19,20]. The ability to rapidly identify AMR to these drugs would be critically useful to veterinarians in the field, who could rapidly adjust their treatment or prevention therapies in response to the detection of such resistance [19]. 

One of the largest challenges in developing our assay was ensuring the concordance between phenotypic AMR status and the detection of genes conferring resistance in complex clinical samples. Investigations of concordance between genotypic and phenotypic resistance based on WGS or selected genes are increasing [21,22], and like the present study, most previous works have found substantial variation in the level of concordance depending on the combination of bacterial species, resistance gene(s) and resistance type. To construct our assay, we selected genes conferring resistance to TETs and MACs that are most commonly found in BRD pathogens and observed good agreement with corresponding phenotypic resistance for some combinations. However, discrepancies between genotypic and phenotypic resistance are not surprising, given the challenges of working with clinical samples outlined above and the large number of potential resistance genes. In one of the most comprehensive databases, ResFinder, more than 140 and 180 genes or gene variants encoding TET or MAC resistance, respectively, have been reported to date [23]. A more comprehensive gene panel may increase the predictive capability of our proposed qPCR assay of resistance in BRD bacterial pathogens. Our in silico analysis showed that the gene targets are potentially carried by other non-BRD pathogen bacteria, which might cause false-positive results if these bacteria were present in sufficient numbers in clinical samples. 

The performance of the PCR-based rapid detection method was most optimal for predicting collective phenotypic resistance among all coexisting BRD pathogens in a single sample. In the MIC test, AMR classification is pathogen dependent, whereas qPCR detects genes in a culture-independent manner regardless of bacterial source. Therefore, a collective phenotypic resistance status for a sample, defined as possessing at least one resistant bacterial pathogen, enables a more rational comparison between the results of MIC testing and qPCR. For predicting phenotypic resistance in individual pathogens, the assay showed the most promise for *M. haemolytica* in lung tissues for both TETs and MACs. Lower agreement was observed for nasal swabs or for *H. somni* isolates, likely due to challenges in the use of the gold-standard method with nasal swab samples and the isolation of *H. somni* from all sample types. Nasal swab samples are frequently contaminated with environmental bacteria, which makes recovery of BRD pathogens challenging. Additionally, because *H. somni* is sensitive to oxygen and a fastidious organism, recovery is low, and false-negative testing results are common. Overall, the results indicate that the newly developed PCR-based rapid detection method is valid for determining the potential likelihood of resistance to TETs and MACs in at least one BRD pathogen or *M. haemolytica*, the most prevalent BRD pathogen, isolated from bovine lung tissues. 

The ROC approach used in our study is in essence a classification method. The gold-standard, culture-based MIC approach gives a dichotomous outcome of resistance or susceptibility to antimicrobials, while the qPCR assay uses a Ct cutoff to transpose the continuous values produced into a finding of resistance or susceptibility. If the purpose of the qPCR assay was to rule out the presence of resistance in a clinical sample for BRD diagnosis based on a susceptible result, a higher Ct cutoff could be used to generate a “rule-out” test with a high Se or low false negatives in detecting resistance. Conversely, a lower Ct cutoff would be more likely to identify infections with the resistance of interest; such a “rule-in” test would have a higher Sp and lower false positives, resulting in higher confidence in the presence of resistance in a sample when a resistant result is observed. In the present study, the ROC approach was used to determine the optimal Ct cutoff by prioritizing a balance between Se and Sp. However, because the qPCR approach provides continuous information, it allows for flexibility in assay design for multiple purposes (such as a priority for “rule-in” or “rule-out” purpose) in real-world scenarios by varying the Ct cutoff.

Although the properties of diagnostic tests are generally expressed by Se and Sp, PPV and NPV are also considered important indicators of a test’s usefulness. Unlike Se and Sp, PPV and NPV depend heavily on prevalence, which in the present study was the prevalence of resistant BRD pathogens determined using the gold-standard method. A low prevalence of AMR would lead to a higher false-positive rate and imply lower PPV, whereas a high prevalence of AMR would lead to a higher false-negative rate and imply lower NPV. In the present study, AMR was more likely to be detected in lung samples than in nasal samples (Table 5), which is largely attributable to the fact that the prevalence of AMR in the samples in this study was lower among nasal samples (Table 3). As a result, the nasal samples had a lower prevalence and PPV, which is the primary reason why the optimal Ct cutoff values obtained for nasal samples were not valid. By contrast, the prevalence of AMR was considerably higher among the lung samples, providing a more balanced outcome in favor of the estimation of Se, Sp, PPV and NPV. Compared with the lung samples, the estimates of Se and Sp for the nasal samples were similar, but PPV was much lower, resulting in invalid estimates of the optimal Ct cutoff values. These results suggest that the most effective application of the qPCR assay for phenotypic AMR classification is situations in which the presence of AMR is not uncommon, such as during BRD outbreaks. 

Although this novel application for predicting AMR phenotypes based on genotypic data could potentially be translated into a rapid assay for use in a variety of platforms, including pen side or in veterinary clinics, several limitations remain to be addressed. First, the data on phenotypic resistance were recategorized into binary outcomes due to the insufficient number of samples with intermediate resistance, and the ROC approach is less optimal for discriminating among more than two classifiers. Second, the classification was conducted for specific pathogen–sample–resistance type combinations in a series of stratified analyses but did not include predictors other than the Ct value, such as historical or clinical information like antimicrobial treatments, which might greatly influence the development of resistance. Third, to estimate the optimal Ct value, the ROC approach employed area under the curve (AUC), which is a widely used methodology but may be further improved because its use of a threshold of 50% for classification ignores the actual probability. To potentially address these limitations and increase the accuracy of diagnosis of AMR among BRD infections using PCR-based rapid detection methods, alternative classification methods, such as the H measure approach, warrant investigation [24]. 

In conclusion, the rapid multiplex real time-based PCR detection assay reported here, which can estimate levels of AMR genes potentially associated with multiple species of BRD pathogens, holds promise for the rapid detection of these genes in complex samples. By combining this gene detection assay with a pathogen-specific assay that can quantify pathogen abundance in samples, veterinarians could rapidly assess the risk of BRD caused by a potentially resistant pathogen, thereby enabling judicious use of antimicrobials in cattle production systems. Further expansion of the number of gene targets may enable more robust AMR assessments of complex samples.

## 4. Materials and Methods

### 4.1. Sample Collection and Distribution

Bovine lung tissue and nasal swab samples were collected over a one-year period in 2018 from submissions, mainly for BRD diagnostic testing, to the University of Nebraska-Lincoln Veterinary Diagnostic Center and combined with an archived sample collection that had been stored at −80 °C from 2012 to 2017. Although detailed clinical information was not available for many submissions, most of these samples were likely collected from non-healthy, clinically ill animals since they were submitted to a veterinary diagnostic lab for BRD diagnosis. A total of 416 bovine clinical samples were collected, primarily consisting of lung tissues (n = 297) and nasal swabs (n = 111). Omitted samples that were excluded from further data analysis were either tissues less related to BRD diagnosis (n = 4, i.e., skin and liver samples) or lacked records about tissue type (n = 4). The samples were analyzed for the existence of target BRD bacterial pathogens. If the pathogens were detected, phenotypic AMR characteristics were subsequently determined using the gold-standard culture-based method. In addition, target genes conferring AMR in the samples were quantified using the culture-independent qPCR rapid detection method developed in this present study. 

### 4.2. Reference Strains

ATCC-sourced strains, a collection of field isolates from diagnostic cases, and off-target controls were used for a preliminary evaluation of the target specificity of the qPCR assay. *M. haemolytica* strains that had been previously subjected to WGS and with established presence or absence of the AMR targets and ICE elements were used to capture the known genomic diversity of this pathogen (Table S3 [19,25]). *H. somni*, *P. multocida*, and *B. trehalosi* strains isolated from clinical cases that had been subjected to MIC testing were also used for evaluation purposes along with ATCC reference strains (Table S4). *Bibersteinia trehalosi* was included in the reference set because it is occasionally isolated from BRD cases and has shown AMR [26]. This collection of reference strains was employed only for evaluating assay performance and specificity and was excluded from further data analysis of the clinical samples. 

### 4.3. Molecular-Based Rapid Detection Assay

**Nucleic acid extraction.** For initial validation, nucleic acids were extracted from reference strains in pure subculture, as described previously [13]. Briefly, a single purified colony was resuspended in nuclease-free water, boiled at 100 °C for 10 min and clarified by centrifugation at 15,700 RCF for 2 min. Swabs, which were submitted to the laboratory in various liquid transport media from referring veterinarians, were vortexed vigorously, and 100 µL of the transport medium was combined with lysis solution and extracted using a MagMax Total Nucleic isolation kit (AM1840) on a Kingfisher flex instrument (Thermo Scientific) following the manufacturer’s instructions. Lung tissue samples were added to a filter Whirl-Pak (Nasco, Fort Atkinson, WI) with 1–5 mL of sterile phosphate-buffered saline (PBS) and placed in a stomacher for 30–60 s. Following stomaching, 2 mL of suspension was removed from the bag, centrifuged at 15,700 RCF for 2 min and extracted using the MagMax procedure and kit, as described above. 

**qPCR assay.** A multiplexed hydrolysis nucleic acid probe assay was designed based on whole-genome sequences of *M. haemolytica* strains possessing ICE elements containing AMR genes. Four gene targets carried on the ICEs were selected, including three that confer resistance to MACs (*msrE*, *mphE*, and *erm42*) and one that is the repressor gene for TET class drug resistance (ICE*tetR*). The primer and probe sequences were designed by using PrimerQuest software (IDT, Iowa City) using genomic sequences from a reference strain of *M. haemolytica* [19] (Table 7). In silico analyses of the primer and probe sequences were conducted to evaluate the use of these targets in addition to *M. haemolytica* strains with the ICEs and four AMR genes and in strains of *P. multocida* and *H. somni*.

Following in silico analysis, primers and probes were purchased from Integrated DNA Technologies (IDT) and used in a single 4-plex reaction in a rotary-based real-time PCR instrument (Qiagen Rotorgene Q). Protocols were optimized for primer and probe concentrations and annealing temperatures. The PCR master mix had a total volume of 25 µL comprising 12.5 μL of 2× Quantifast Multiplex PCR Master Mix (Qiagen), 1 μL of each primer probe mix (4 μL total) containing F (10 μM), R (10 μM), and P (10 μM), 6.5 μL of nuclease-free water and 2 µL of template DNA. The thermocycling conditions were as follows: 95 °C for 5 min, followed by 45 cycles of 95 °C for 15 s, and 59 °C for 40 s. The Ct threshold was set at a fixed value of 0.1 for all detection channels following dynamic tube normalization.

Following optimization, assay performance was tested using reference strains, and limit of detection analysis was conducted using a reference *M. haemolytica* strain known through WGS to have all four genes (Reference strain 1621 in Table S3) [19]. The assay was further validated using a panel of *M. haemolytica* strains (Table S3) previously characterized by WGS and representing all known major genotypes and subtypes of *M. haemolytica* and different combinations of AMR genes [19]. The assay gene targets have also been reported in strains of *H. somni* and *P. multocida* isolated from cattle, and therefore strains of *H. somni* and *P. multocida* with phenotypic resistance patterns similar to those of the *M. haemolytica* strain collection were also included in this validation (Table S4). In addition, analyses of non-target closely related strains, including *B. trehalosi*, and other ATCC reference strains were performed (Table S4).

### 4.4. Preliminary Assay Validation and Analytical Sensitivity

Assay performance was evaluated for all four targets using DNA extracted from *M. haemolytica* reference strain 1621. Briefly, the strain was grown in pure culture on tryptic soy agar with 5% sheep blood (BAP) (Remel, Lenexa, KS). Colonies were picked and cultured in 10 mL of brain heart infusion broth in a 100 mL flask shaken at 200 RPM for 12 h. Serial dilutions (1:10) of this culture were plated on BAP to enumerate colonies and estimate colony forming units/mL, and the remaining portion of each dilution was used for nucleic acid extraction, as described above, to evaluate assay performance and limit of detection (Table 1). Following the determinations of limit of detection and dynamic range, the target specificity of the assay was evaluated using a diverse collection of reference strains and controls (Table S4).

### 4.5. Pathogen Isolation and Determination of Phenotypic Antimicrobial Resistance Characteristics

**BRD pathogen isolation and confirmation**. All clinical samples were processed for bacterial isolation and identification of the BRD pathogens *M. haemolytica*, *P. multocida*, and *H. somni*, as described previously [13]. Briefly, samples were documented, processed, and tested by trained personnel following validated and approved standard operating procedures in an American Association of Veterinary Laboratory Diagnosticians (AAVLD)-accredited diagnostic laboratory. For bacterial culture, diseased portions of fresh lung tissues were excised using flame-sterilized scissors and dipped in alcohol. After flame sterilizing the exterior, the sample was bisected, and the cut surface was used to inoculate culture media. Swabs were used directly to inoculate plates if submitted in liquid medium or otherwise were resuspended in approximately 1.5 mL of PBS. Colonies were isolated by streaking on culture media, including BAP, chocolate agar, and MacConkey agar (Remel, Lenexa, KS), incubated in 5% CO_2_ and examined at 24 and 48 h after inoculation. Colonies with morphologies consistent with *M. haemolytica*, *P. multocida*, or *H. somni* were identified by MALDI-TOF MS (Bruker Biotyper) testing using the manufacturer’s validated procedures for definitive identification of these organisms.

**MIC determination**. For each confirmed BRD pathogen strain, the presence of phenotypic AMR was evaluated using oxytetracycline to represent the TET class and tilmicosin and tulathromycin to represent the MAC class. As the gold-standard approach, AMR to TETs and MACs was evaluated by MIC testing using the broth microdilution method according to CLSI guidelines [27]. By comparing the MIC values with the corresponding breakpoints for bovine respiratory pathogens (Thermo Sensititre using the BOPO6F panel), which are listed in Table 4, BRD pathogen isolates were classified as S, I or R to each drug [28]. In this study, intermediate and resistant were both re-defined as “resistant” (R+I) at the strain level. A sample was classified as containing “resistant” BRD pathogens if at least one strain of antimicrobial-resistant BRD pathogens evaluated in this study was detected.

### 4.6. Predicting Phenotypic Antimicrobial Resistance

Statistical analysis was performed to optimize and validate the capability of the developed qPCR assay to classify samples as either S or R+I to a given antimicrobial. First, optimal Ct cutoff values were determined using ROC analysis to maximize the assay accuracy (both Se and Sp as the primary accuracy indices) compared to the gold-standard test. Optimal cutoff values were determined for clinical samples containing *M. haemolytica*, *P. multocida* or *H. somni* resistant to OTC, tilmicosin or tulathromycin, as well as for samples classified as “possessing at least one resistant BRD pathogen”. Second, the diagnostic accuracy and validity of the developed qPCR assay were assessed by determining the sufficiency of the sample size collected in this study for detecting MAC and TET resistance in samples possessing BRD pathogens and by using the cross-validation technique. All statistical analyses were performed in statistical software R version 3.5.1 [29].

#### 4.6.1. Optimal Ct Cutoff Value Determination

**Data preparation for ROC analysis.** To determine the optimal Ct, ROC curves were plotted by comparing the phenotypic AMR classification determined by the gold-standard test with the continuous Ct value generated by the qPCR assay using the ‘pROC’ package in statistical software R [30]. ROC curve analysis is a common method for evaluating diagnostic tests with a binary classifier [31], but this method does not work optimally if there is no detection or anomaly observed in either the qPCR assay or MIC test. In this study, no detection by qPCR means that the target gene is either present in very small amounts or completely absent. Hence, samples recorded as “no gene copies detected” were imputed with a Ct value of 40, the highest amplification cycle observed in this study. For the gold-standard test, it is not easy to reinvestigate missing results. Hence, another four samples were further excluded from data analysis due to a lack of MIC records, resulting in a total of 404 samples (296 lung and 108 nasal) for inclusion in the following data analysis. 

**ROC analysis to determine the optimal Ct cutoff.** In this study, ROC curves were generated by plotting the true-positive rate (i.e., Se) against the false-positive rate (i.e., 1–Sp) to determine the diagnostic equivalency between the qPCR assay and the gold-standard test over different cutoff values. Table 8 shows a schematic representation of the comparison of the outcomes of the qPCR assay and the culture-based gold-standard test and the calculation of Se and Sp. The optimal cutoff translating the continuous Ct value into a binary classification of R+I or S was determined by optimizing the AUC [32]. AUC can take values between 0.5 and 1, with values closer to 1 indicating high performance of the qPCR approach in predicting the resistance classification with minimum classification error compared with the gold-standard test.

**Performance of qPCR.** As a post-analysis statistic, the determined optimal Ct cutoff value was used to calculate Cohen’s Kappa (κ) in order to measure the agreement between the two tests [33]. κ ranges from −1 to 1, with higher values implying greater concordance between the two tests. The following interpretations of κ were used: κ < 0.2, poor agreement; 0.2 < κ ≤ 0.4, fair agreement; 0.4 < κ ≤ 0.6, moderate agreement; 0.6 < κ ≤ 0.8, good agreement; and κ > 0.8, very good agreement [34]. κ was calculated using Equation (1):

(1) $$\begin{array}{c} \kappa =\frac{Actual\;agreement\;beyond\;chance\;}{Potential\;agreement\;beyond\;chance\;}=\frac{p_{0}-p_{e}}{1-p_{e}},\\ where\;p_{0}=\;proportion\;of\;true\;agreement=\frac{TP+TN}{TP+FN+FP+TN}\;and\\ p_{e}=proportion\;of\;random\;agreement=\frac{TPFN*TPFP+FPTN*FNTN}{{\left(TP+FN+FP+TN\right)}^{2}},\\ TPFN=TP+FN,\;TPFP=TP+FP,\;FPTN=FP+TN\;and\;FNTN=FN+TN,\\ TP,\;TN,\;FN\;and\;FP\;are\;reported\;in\;\mathrm{Table}\;8. \end{array}$$

The PPV and NPV were also calculated to measure the true-positive and true-negative results of the multiplex qPCR assay, as shown in Table 8. In this study, PPV is the proportion of samples showing a test result of “resistant” based on the multiplex qPCR assay that were actually identified as resistant using the gold-standard test, while NPV is the proportion of samples showing a test result of “susceptible” in the qPCR assay that were also identified as susceptible using the gold-standard test.

#### 4.6.2. Diagnostic Accuracy Evaluation

Developing a multiplex qPCR assay with relatively high diagnostic accuracy for classifying AMR status in clinical samples is contingent on a balance of samples with positive and negative responses to the outcomes of interest, i.e., R+I and S [35]. To determine whether this balance was reached, the minimum required sample size and the required proportion of resistant samples were estimated and compared with the sample sizes and the proportions of resistant samples included in this study to validate the optimal Ct cutoff values determined using the ROC approach. Once the sufficiency of the sample size and required number of resistant samples were determined to be adequate, cross-validation (CV) was conducted to evaluate the computational approach used in ROC analysis to determine the optimal Ct value. Cross-validation is one of the most common resampling methods to evaluate the performance of a diagnostic test [36]. Here, the 5-fold CV technique was used to validate the optimal Ct value of the multiplex qPCR assay for classifying AMR equivalent to the gold standard. Further details of the accuracy evaluation techniques are provided in the Supplementary Materials, Text.

## Figures and Tables

**Table 1:** Sensitivity and limit of detection in colony forming units per reaction (CFU/rxn) using a *Mannheimia haemolytica* reference strain (1621) confirmed to have targets by whole-genome sequencing.

Target	CFU/rxn	Ct ^1^	CD ^2^	RE ^3^
ICE*TetR*	3200	26.78		
ICE*TetR*	320	30.23		
ICE*TetR*	32	33.53		
ICE*TetR*	3.2	37.34	0.982	0.95
*erm42*	3200	26.32		
*erm42*	320	29.85		
*erm42*	32	32.95		
*erm42*	3.2	36.17	0.986	1.00
*mph E*	3200	25.61		
*mph E*	320	29.05		
*mph E*	32	32.30		
*mph E*	3.2	36.56	0.985	0.88
*msr E*	3200	25.44		
*msr E*	320	28.82		
*msrE*	32	32.04		
*msr E*	3.2	35.70	0.985	0.98

^1^ Ct = threshold cycle, ^2^ CD = correlation of determination, R^2^, ^3^ RE = reaction efficiency (10^(−1/slope)−1).

**Table 2:** Sample distribution by clinical sample type and pathogen.

Sample Type ^1^	Total Sample Size ^2^	Occurrence of BRD Pathogens No. of Positive Samples (Prevalence, 95% CI)			
		*M. haemolytica*	*P. multocida*	*H. somni*	Positive for at Least One BRD Pathogen
Lung sample	297	191 (64.3%, 58.7–69.5%)	96 (32.3%, 27.3–37.8%)	93 (31.3%, 26.3–36.8%)	296 (99.7%, 98.1–100.0%)
Nasal sample	111	72 (64.9%, 55.6–73.1%)	80 (72.1%, 63.1–79.6%)	29 (26.1%, 18.9–35.0%)	108 (97.3%, 92.4–99.1%)
Others—skin/liver	4	1 (25.0%, 4.6–69.9%)	0 (0.0%, 0.0%–49.0%)	1 (25.0%, 4.6–69.9%)	2 (50%, 15.0–85.0%)
Missing	4	1 (25.0%, 4.6–69.9%)	1 (25.0%, 4.6–69.9%)	1 (25.0%, 4.6–69.9%)	1 (25.0%, 4.6–69.9%)
Total	416	265 (63.7%, 59.0–68.2%)	177 (42.5%, 37.9–47.3%)	123 (29.6%, 25.4–34.1%)	407 (97.8%, 95.9–98.8%)

^1^ Lung samples refer to lung tissues and abdominal fluid clinical samples; nasal samples are collected as nasal swabs; other samples are either skin or liver tissues; missing samples are those lacking records about tissue types. ^2^ The distribution of the samples by the pathogens is not exclusive, as more than one pathogen can be isolated from a single sample.

**Table 3:** Summary of minimum inhibitory concentration test results by sample type, pathogen, and antimicrobial.

Pathogen	Sample	Class	Antibiotics	No. of Total Samples	No. (Percentage) of Samples with ^1^			
					R	I	R+I	S
*M. haemolytica*	Lung	Tetracycline	Oxytetracycline	191	78 (40.8%)	3 (1.6%)	81 (42.4%)	110 (57.6%)
		Macrolide	Tilmicosin	191	69 (36.1%)	5 (2.6%)	74 (38.7%)	117 (61.3%)
		Macrolide	Tulathromycin	191	64 (33.5%)	3 (1.6%)	67 (35.1%)	124 (64.9%)
		Macrolide	Tilmicosin or tulathromycin	191	75 (39.3%)	0 (0%)	75 (39.3%)	116 (60.7%)
	Nasal	Tetracycline	Oxytetracycline	72	3 (4.2%)	2 (2.7%)	5 (6.9%)	67 (93.1%)
		Macrolide	Tilmicosin	72	2 (2.8%)	0 (0%)	2 (2.8%)	70 (97.2%)
		Macrolide	Tulathromycin	72	6 (8.3%)	0 (0%)	6 (8.3%)	66 (91.7%)
		Macrolide	Tilmicosin or tulathromycin	72	6 (8.3%)	0 (0%)	6 (8.3%)	66 (91.7%)
*P. multocida*	Lung	Tetracycline	Oxytetracycline	96	28 (29.2%)	2 (2.1%)	30 (31.3%)	66 (68.7%)
		Macrolide	Tilmicosin	95	15 (15.8%)	1 (1.0%)	16 (16.8%)	79 (83.2%)
		Macrolide	Tulathromycin	96	9 (9.4%)	1 (1.1%)	10 (10.5%)	86 (89.5%)
		Macrolide	Tilmicosin or tulathromycin	95	15 (15.8%)	1 (1.0%)	16 (16.8%)	79 (83.2%)
	Nasal	Tetracycline	Oxytetracycline	78	15 (19.2%)	0 (0%)	15 (19.2%)	63 (80.8%)
		Macrolide	Tilmicosin	80	6 (7.5%)	0 (0%)	6 (7.5%)	74 (92.5%)
		Macrolide	Tulathromycin	78	1 (1.3%)	3 (3.8%)	4 (5.1%)	74 (94.9%)
		Macrolide	Tilmicosin or tulathromycin	80	7 (8.8%)	0 (0%)	7 (8.8%)	73 (91.2%)
*H. somni*	Lung	Tetracycline	Oxytetracycline	93	41 (44.1%)	8 (8.6%)	49 (52.7%)	44 (47.3%)
		Macrolide	Tilmicosin	93	20 (21.5%)	1 (1.1%)	21 (22.6%)	72 (77.4%)
		Macrolide	Tulathromycin	93	20 (21.5%)	8 (8.6%)	28 (30.1%)	65 (69.9%)
		Macrolide	Tilmicosin or tulathromycin	93	26 (28.0%)	6 (6.4%)	32 (34.4%)	61 (65.6%)
	Nasal	Tetracycline	Oxytetracycline	29	13 (44.8%)	0 (0%)	13 (44.8%)	16 (55.2%)
		Macrolide	Tilmicosin	29	2 (6.9%)	1 (3.4%)	3 (10.3%)	26 (89.7%)
		Macrolide	Tulathromycin	28	5 (17.9%)	2 (7.1%)	7 (25.0%)	21 (75.0%)
		Macrolide	Tilmicosin or tulathromycin	28	6 (21.4%)	3 (10.7%)	9 (32.1%)	19 (67.9%)
At least one BRD pathogen	Lung	Tetracycline	Oxytetracycline	296	124 (41.9%)	8 (2.7%)	132 (44.6%)	164 (55.4%)
		Macrolide	Tilmicosin	295	95 (32.2%)	6 (2.0%)	101 (34.2%)	194 (65.8%)
		Macrolide	Tulathromycin	296	87 (29.4%)	8 (2.7%)	95 (32.1%)	197 (67.9%)
		Macrolide	Tilmicosin or tulathromycin	295	105 (35.6%)	4 (1.3%)	109 (36.9%)	182 (63.1%)
	Nasal	Tetracycline	Oxytetracycline	108	23 (21.3%)	2 (1.8%)	25 (23.1%)	83 (76.9%)
		Macrolide	Tilmicosin	108	11 (10.2%)	0 (0.0%)	11 (10.2%)	97 (89.8%)
		Macrolide	Tulathromycin	108	11 (10.2%)	5 (4.6%)	16 (14.8%)	92 (85.2%)
		Macrolide	Tilmicosin or tulathromycin	108	19 (17.6%)	2 (1.8%)	21 (19.4%)	87 (80.6%)

^1^ S, R, I denotes susceptible, resistant, and intermediate resistant to the drug classified based on MIC test and CLSI breakpoints, and the categories of R and I are combined into a new category of “resistant”, or R+I.

**Table 4:** Phenotypic classification of antimicrobial resistance based on minimum inhibitory concentration measurements by antibiotics tested in this study.

Class	Antibiotics	Antimicrobial Resistance Classification			
		Susceptible (S)	Intermediate (I)	Resistant (R)	“Resistant” (R+I)
**Tetracycline**	Oxytetracycline	≤2	>2 and ≤8	>8	>2
**Macrolide**	Tilmicosin	≤8	>8 and ≤32	>32	>8
**Macrolide**	Tulathromycin	≤16	>16 and ≤32	>32	>16

**Table 5:** Optimal cycle threshold (Ct) cutoff value by sample type and antibiotics.

BRD Pathogen	Sample	Class	Antibiotics	No. of Total Samples	No. of Samples with ^1^		Optimal Cycle Threshold (Ct)	Se ^2^ (%)	Sp ^2^ (%)	AUC ^2^ (%)	Kappa (*κ*)	Prevalence (%)	PPV ^2^ (%)	NPV ^2^ (%)
					R+I	S								
*M. haemolytica*	Lung	Tetracycline	Oxytetracycline	191	81	110	31.00	77.78	95.45	89.99	0.75	42.41	92.65	85.37
		Macrolide	Tilmicosin	191	74	117	33.04	74.32	93.16	86.14	0.69	38.74	87.30	85.16
		Macrolide	Tulathromycin	191	67	124	32.89	79.10	92.74	88.94	0.73	35.08	85.48	89.15
		Macrolide	Tilmicosin or tulathromycin	191	75	116	33.04	73.33	93.10	85.45	0.68	39.27	87.30	84.38
	Nasal	Tetracycline	Oxytetracycline	72	5	67	32.26	100.00	79.10	92.54	0.34	6.94	21.83	98.35
		Macrolide	Tilmicosin	72	2	70	21.42	100.00	100.00	100.00	1.00	2.78	52.25	99.17
		Macrolide	Tulathromycin	72	6	66	30.73	50.00	86.36	63.89	0.25	8.33	25.00	95.00
		Macrolide	Tilmicosin or tulathromycin	72	6	66	30.73	50.00	86.36	63.89	0.25	8.33	25.00	95.00
*P. multocida*	Lung	Tetracycline	Oxytetracycline	96	30	66	36.10	83.33	86.36	86.52	0.67	31.25	73.53	91.94
		Macrolide	Tilmicosin	95	16	79	32.91	56.25	89.87	72.23	0.45	16.84	52.94	91.03
		Macrolide	Tulathromycin	96	10	86	32.91	80.00	89.53	84.94	0.53	10.42	47.06	97.47
		Macrolide	Tilmicosin or tulathromycin	95	16	79	32.91	56.25	89.87	72.23	0.45	16.84	52.94	91.03
	Nasal	Tetracycline	Oxytetracycline	78	15	63	29.35	66.67	92.06	88.04	0.59	19.23	66.67	92.06
		Macrolide	Tilmicosin	80	6	74	36.40	66.67	62.16	59.01	0.10	7.50	12.50	95.83
		Macrolide	Tulathromycin	78	4	74	31.47	50.00	78.38	58.78	0.11	5.13	11.11	96.67
		Macrolide	Tilmicosin or tulathromycin	80	7	73	32.22	42.86	76.71	56.36	0.11	8.75	15.00	93.33
*H. somni*	Lung	Tetracycline	Oxytetracycline	93	49	44	36.28	81.63	72.73	75.72	0.55	52.69	76.92	78.05
		Macrolide	Tilmicosin	93	21	72	33.08	61.90	79.17	67.29	0.37	22.58	46.43	87.69
		Macrolide	Tulathromycin	93	28	65	31.67	60.71	87.69	75.36	0.50	30.11	68.00	83.82
		Macrolide	Tilmicosin or tulathromycin	93	32	61	33.08	62.50	86.89	76.95	0.51	34.41	71.43	81.54
	Nasal	Tetracycline	Oxytetracycline	29	13	16	32.85	92.31	56.25	62.50	0.47	44.83	63.16	90.00
		Macrolide	Tilmicosin	29	3	26	30.88	66.67	65.38	52.56	0.15	10.34	18.18	94.44
		Macrolide	Tulathromycin	28	7	21	27.19	100.00	38.10	68.03	-0.36	25.00	10.54	67.85
		Macrolide	Tilmicosin or tulathromycin	28	9	19	26.83	100.00	36.84	66.67	−0.39	32.14	12.45	59.49
At least one BRD pathogen	Lung	Tetracycline	Oxytetracycline	296	132	164	36.06	81.06	82.93	85.29	0.64	44.59	79.26	84.47
		Macrolide	Tilmicosin	295	101	194	33.08	69.31	89.69	81.25	0.61	34.24	77.78	84.88
		Macrolide	Tulathromycin	296	95	201	32.89	72.63	90.55	84.29	0.64	32.09	78.41	87.50
		Macrolide	Tilmicosin or tulathromycin	295	109	186	33.08	67.89	91.40	81.94	0.62	36.95	82.22	82.93
	Nasal	Tetracycline	Oxytetracycline	108	25	83	32.81	88.00	79.52	90.07	0.56	23.15	56.41	95.65
		Macrolide	Tilmicosin	108	11	97	31.82	63.64	82.47	71.42	0.30	10.19	29.17	95.24
		Macrolide	Tulathromycin	108	16	92	31.47	43.75	83.70	57.54	0.24	14.81	31.82	89.53
		Macrolide	Tilmicosin or tulathromycin	108	21	87	31.82	42.86	82.76	57.85	0.24	19.44	37.50	85.71

^1^ S, R, I denotes susceptible, resistant, and intermediate resistant to the drug classified based on MIC test and CLSI breakpoints, and the categories of R and I are combined into a new category of “resistant”, or R+I. ^2^ Se = sensitivity; Sp = specificity; AUC = area under the curve; PPV = positive predictive value; NPV = negative predictive value.

**Table 6:** Optimal cycle threshold (Ct) cutoff value for lung and nasal samples.

Class	Antibiotics	Lung Sample		Nasal Sample	
		Optimal Cycle Threshold (Ct)	Kappa (*κ*)	Optimal Cycle Threshold (Ct)	Kappa (*κ*)
Tetracycline	Oxytetracycline	35.66	0.61	33.27	0.49
Macrolide	Tilmicosin	33.12	0.61	-	-
Macrolide	Tulathromycin	32.64	0.63	-	-
Macrolide	Tilmicosin or tulathromycin	33.12	0.62	-	-

**Table 7:** Oligonucleotide sequences for primers and probes used in the assay.

Target	Primer/Probe	Sequence (5′-3′)	Size	Reference
*tetR*	ICE*tetR*-F	TTTGGCTTTCTTGATGCTCTTG	71	This paper
	ICE*tetR*-R	GTGATGCTGGGTTTAGTCTATCT		
	ICE*tetR*-P (CY5/TAO-IAB-RQ)	CGCAATAGAGCTTAATGCATACACGGC		
*erm42*	*erm42*-F	GCCGTTAATGCTATTGAGTTCG	105	This paper
	*erm42*-R	CGGCTTCAATAATAGACACATTTGA		
	*erm42*-P (FAM/ZEN-IAB-FQ)	AGTGTATTGGCTGATAAGTTGAGCCATGA		
*msrE*	*msrE*-F	GGGTGGTTACTCGGATTACTTG	88	This paper
	*msrE*-R	CTCCCGTTCCTTCATCATCAG		
	*msrE*-P (Texas Red/IAB-RQ)	AGCGACAACACCAAGCCGTAGAAT		
*mphE*	*mphE*-F	TTGGAAACCCGCTACAGAAA	113	This paper
	*mphE*-R	GCTCCATCCTTTGAAGCTAGT		
	*mphE*-P (JOE/ZEN-IAB-FQ)	TGATGTTCTATGGGCAGATTTCACCCA		

**Table 8:** Schematic representation of the outcomes of the multiplex qPCR assay detecting antimicrobial resistance compared to the gold standard.

Multiplex qPCR Assay	Culture-Based Gold-Standard Test		
	“R” ^1^	S ^2^	
“R” ^1^	True Positive (TP)	False Positive (FP)	PPV ^3^ = TP/(TP + FP)
S ^2^	False Negative (FN)	True Negative (TN)	NPV ^3^ = T N/(TN + FN)
	Se ^3^ = TP/(TP + FN)	Sp ^3^ = TN/(TN + FP)	

^1^ “R” indicated by the gold-standard test refers to samples containing pathogens resistant (R) or intermediate resistant (I) to antibiotics tested (“resistant” or R+I); hence classification given qPCR results based on the comparison to the gold-standard test also refers to the redefined “resistant” in this study. ^2^ S indicated by both the gold-standard test and qPCR assay refers to samples containing pathogens susceptible to antibiotics tested. ^3^ Se = sensitivity; Sp = specificity; PPV = positive predictive value; and NPV = negative predictive value.

## Data Availability

All data are available in the main text or as electronic supplementary.

## Supplementary Materials

The following are available online at https://www.mdpi.com/2076-0817/10/1/64/s1, Table S1: *M. haemolytica* in silico primer and probes analysis; Table S2: Primer binding other species; Table S3: Isolates used in MALDI study (*M. haemolytica* WGS isolates); Table S4: Target and non-target isolates used to validate assay specificity, with respective threshold cycle (Ct) values obtained from multiplex qPCR and MIC values for antimicrobial resistance determination; Table S5: Assessment of sufficiency of sample size for determining the optimal cycle threshold value; Table S6: Comparison of 5-fold cross-validation with the optimal threshold cycle (Ct) cutoff value obtained using ROC curves on the overall data; Text: Elaboration of accuracy evaluation techniques used in the present study.
