# Supplementary material for: Development of a Multiplex Real-Time PCR Assay for Predicting Macrolide and Tetracycline Resistance Associated with Bacterial Pathogens of Bovine Respiratory Disease
Source: Pathogens. 2021 Jan 13;10(1):64. doi: 10.3390/pathogens10010064 (PMC7828349; doi:10.3390/pathogens10010064)
Supplement: Supplementary file 1 [file pathogens-10-00064-s001.zip › Supplementary materials - Table S2_S4_S5_S6_R1.docx]

**Supplementary materials - Table S2**. Primer binding other species.

| Accession | F_coverage_ | F_start_ | F_end_ | P_coverage_ | P_start_ | P_end_ | R_coverage_ | R_start_ | R_end_ |
| --- | --- | --- | --- | --- | --- | --- | --- | --- | --- |
| CP047349.1 | 100 | 3188078 | 3188099 | 95.833 | 3188130 | 3188153 | 95.455 | 3188182 | 3188161 |
| CP034668.1 | 100 | 3395406 | 3395427 | 95.833 | 3395458 | 3395481 | 95.455 | 3395510 | 3395489 |
| CP045008.1 | 100 | 3373133 | 3373154 | 95.833 | 3373185 | 3373208 | 95.455 | 3373237 | 3373216 |
| MK134853.1 | 100 | 2781 | 2802 | 100 | 2833 | 2859 | 100 | 2885 | 2863 |
| MK110805.1 | 100 | 2853 | 2874 | 100 | 2905 | 2931 | 100 | 2957 | 2935 |
| CP026856.1 | 100 | 2611214 | 2611235 | 100 | 2611266 | 2611292 | 100 | 2611318 | 2611296 |
| CP026860.1 | 100 | 316468 | 316489 | 100 | 316520 | 316546 | 100 | 316572 | 316550 |
| CP024444.1 | 100 | 151028 | 151049 | 100 | 151080 | 151106 | 100 | 151132 | 151110 |
| CP011374.1 | 100 | 2160774 | 2160795 | 100 | 2160826 | 2160852 | 100 | 2160878 | 2160856 |
| KJ909292.1 | 100 | 4560 | 4581 | 100 | 4612 | 4638 | 100 | 4664 | 4642 |
| KC734562.1 | 100 | 3761 | 3782 | 100 | 3813 | 3839 | 100 | 3865 | 3843 |
| KC734560.1 | 100 | 3861 | 3882 | 100 | 3913 | 3939 | 100 | 3965 | 3943 |
| HF953351.1 | 100 | 10357 | 10378 | 100 | 10409 | 10435 | 100 | 10461 | 10439 |
| CP003745.1 | 100 | 83569 | 83590 | 100 | 83621 | 83647 | 100 | 83673 | 83651 |
| CP003745.1 | 100 | 83569 | 83590 | 100 | 83621 | 83647 | 100 | 107554 | 107532 |
| CP003745.1 | 100 | 83569 | 83590 | 100 | 107502 | 107528 | 100 | 83673 | 83651 |
| CP003745.1 | 100 | 83569 | 83590 | 100 | 107502 | 107528 | 100 | 107554 | 107532 |
| CP003745.1 | 100 | 107450 | 107471 | 100 | 83621 | 83647 | 100 | 83673 | 83651 |
| CP003745.1 | 100 | 107450 | 107471 | 100 | 83621 | 83647 | 100 | 107554 | 107532 |
| CP003745.1 | 100 | 107450 | 107471 | 100 | 107502 | 107528 | 100 | 83673 | 83651 |
| CP003745.1 | 100 | 107450 | 107471 | 100 | 107502 | 107528 | 100 | 107554 | 107532 |
| AM992204.1 | 100 | 4183 | 4204 | 100 | 4235 | 4261 | 100 | 4287 | 4265 |
| AY987962.1 | 100 | 658 | 679 | 100 | 710 | 736 | 100 | 762 | 740 |
| AJ245947.1 | 100 | 429 | 450 | 100 | 481 | 507 | 100 | 533 | 511 |
| AY362554.1 | 100 | 2898 | 2919 | 100 | 2950 | 2976 | 100 | 3002 | 2980 |
| CP053042.1 | 100 | 2567543 | 2567522 | 95.833 | 2567491 | 2567468 | 95.455 | 2567439 | 2567460 |
| CP053044.1 | 100 | 573063 | 573042 | 95.833 | 573011 | 572988 | 95.455 | 572959 | 572980 |
| CP047639.1 | 100 | 1269079 | 1269058 | 100 | 1269027 | 1269001 | 100 | 1268975 | 1268997 |
| CP047639.1 | 100 | 2711671 | 2711685 | 100 | 1269027 | 1269001 | 100 | 1268975 | 1268997 |
| CP047340.1 | 100 | 124594 | 124573 | 95.833 | 124542 | 124519 | 95.455 | 124490 | 124511 |
| KX426227.1 | 100 | 83943 | 83922 | 100 | 83891 | 83865 | 100 | 83839 | 83861 |
| CP006956.1 | 100 | 241917 | 241896 | 100 | 241865 | 241839 | 100 | 241813 | 241835 |
| CP006955.1 | 100 | 2348525 | 2348504 | 100 | 2348473 | 2348447 | 100 | 2348421 | 2348443 |
| CP006955.1 | 100 | 2348525 | 2348504 | 100 | 2348473 | 2348447 | 100 | 2372260 | 2372282 |
| CP006955.1 | 100 | 2348525 | 2348504 | 100 | 2372312 | 2372286 | 100 | 2348421 | 2348443 |
| CP006955.1 | 100 | 2348525 | 2348504 | 100 | 2372312 | 2372286 | 100 | 2372260 | 2372282 |
| CP006955.1 | 100 | 2372364 | 2372343 | 100 | 2348473 | 2348447 | 100 | 2348421 | 2348443 |
| CP006955.1 | 100 | 2372364 | 2372343 | 100 | 2348473 | 2348447 | 100 | 2372260 | 2372282 |
| CP006955.1 | 100 | 2372364 | 2372343 | 100 | 2372312 | 2372286 | 100 | 2348421 | 2348443 |
| CP006955.1 | 100 | 2372364 | 2372343 | 100 | 2372312 | 2372286 | 100 | 2372260 | 2372282 |
| CP006942.1 | 100 | 975286 | 975265 | 100 | 975234 | 975208 | 100 | 975182 | 975204 |
| KC734561.1 | 100 | 4169 | 4148 | 100 | 4117 | 4091 | 100 | 4065 | 4087 |
| FJ012880.1 | 100 | 36507 | 36486 | 100 | 36455 | 36429 | 100 | 36403 | 36425 |

**Supplementary materials - Table S4**. Target and non-target isolates used to validate assay specificity, with respective cycle threshold (Ct) values obtained from multiplex qPCR and MIC values for antimicrobial resistance determination.

| Isolate (Accession Number) | Species | Antibiotic Susceptibility^1^ | | MIC Values (µg/mL) | | Cycle threshold (Ct) Values^2^ | | | |
| --- | --- | --- | --- | --- | --- | --- | --- | --- | --- |
|  |  | Macrolide | Tetracycline | Tulathromycin | Tetracycline | *erm42* | *msrE* | *mphE* | ICE*tetR* |
| 2016003312 | *H. somni* | R | S | >64 | 4 |  |  |  | 16.72 |
| 2016003129 | *H. somni* | R | R | >64 | 8 | 15.47 |  |  | 17.21 |
| 2016005177 | *H. somni* | R | R | 64 | >8 | 13.54 | 12.6 | 13.37 | 17.48 |
| 2016004379 | *H. somni* | R | R | >64 | 8 | 14.19 | 12.71 | 13.59 | 15.81 |
| 2018004447 | *H. somni* | R | R | 64 | >8 | 14.56 | 13.24 | 14.05 | 16.21 |
| 2018003024 | *H. somni* | R | R | >64 | >8 |  |  |  | 16.71 |
| 2018005809 | *H. somni* | R | R | >64 | 8 | 14.66 | 13.38 | 14.2 | 16.29 |
| 2018011103 | *H. somni* | R | R | >64 | >8 |  | 11.91 | 12.42 | 17.99 |
| 2019001055 | *H. somni* | R | R | 32 | >8 | 15.51 | 14.29 | 15.22 | 17.33 |
| 2019001996 | *H. somni* | R | R | >64 | >8 | 15.23 | 13 | 13.95 | 16.74 |
| 2016000948 | *H. somni* | R | S | >64 | <=0.5 | 12.38 | 13.31 | 14.12 | 16.65 |
| 2015003760 | *H. somni* | R | S | >64 | 4 |  |  |  | 17.01 |
| 2019002695 | *H. somni* | R | R | >64 | >8 | 15.25 | 14.01 | 14.89 | 16.92 |
| 2016004313 | *H. somni* | R | S | >64 | <=0.5 |  |  |  | 15.76 |
| 2016003735 | *H. somni* | R | S | 64 | <=0.5 | 14.52 | 13.23 | 14.15 | 16.31 |
| 2018010157 | *H. somni* | R | S | >64 | 1 | 13.11 | 14.27 | 15.11 | 17.29 |
| 2019001152 | *H. somni* | R | R | 32 | >8 | 14.61 | 13.29 | 14.19 | 16.27 |
| 2019005019 | *H. somni* | R | R | 32 | >8 | 12.32 |  | 17.54 | 26.59 |
| 2019003774 | *H. somni* | R | R | >64 | >8 |  |  |  | 21.15 |
| 2018007471 | *H. somni* | R | R | 64 | >8 | 14.61 |  | 17.11 | 25.96 |
| 2019005004 | *H. somni* | R | R | >64 | >8 |  |  |  | 20.15 |
| 2018004588 | *H. somni* | R | R | 64 | >8 | 18.68 |  | 16.97 | 25.65 |
| 2018008918 | *H. somni* | S | S | 8 | 1 |  |  |  |  |
| 2018006051 | *H. somni* | S | S | 2 | <=0.5 |  |  |  |  |
| 2019000908 | *H. somni* | S | S | 4 | 1 |  |  |  |  |
| 2019003011 | *H. somni* | S | R | 4 | >8 |  |  |  | 16.36 |
| 2019000681 | *H. somni* | S | S | 2 | 1 |  |  |  |  |
| 2017003091 | *H. somni* | S | S | 4 | <=0.5 |  |  |  |  |
| 2015004975 | *H. somni* | S | S | <=1 | <=0.5 |  |  |  |  |
| 2014010339 | *H. somni* | S | R | 4 | >8 |  |  |  | 16.77 |
| 2015004145 | *H. somni* | S | S | <=1 | <=0.5 |  | 39.24 |  |  |
| UNL 2018000848 | *H. somni* | R | S | >64 | 2 |  |  |  | 15.65 |
| UNL 2018001358 | *H. somni* | R | R | >64 | >8 |  |  |  | 21.94 |
| UNL 2018001311 | *H. somni* | S | S | 2 | <0.5 |  |  |  |  |
| UNL 2018001868 | *H. somni* | S | S | <1 | <0.5 |  |  |  |  |
| UNL 2018001110 | *P. multocida* | S | S | <=1 | <=0.5 |  |  |  |  |
| UNL 2018001311 | *P. multocida* | S | S | <=1 | <=0.50 |  |  |  |  |
| UNL 2018001309 | *P. multocida* | R | R | 32 | >8 | 16.48 | 15.08 | 15.41 | 16.43 |
| UNL 2018001126 | *P. multocida* | S | S | 2 | 1 |  |  |  |  |
| UNL 2018001132 | *P. multocida* | S | S | 4 | <=0.5 |  |  |  |  |
| UNL 2018001126 | *Bibersteinia trehalosi* | S | S | 2 | 1 |  |  |  |  |
| UNL 2018003753 | *Bibersteinia trehalosi* | R | R | 64 | >8 |  | 13.23 | 13.71 | 14.67 |
| ATCC 49244 | *Mannheimia granulomatis* | NA | NA | NA | NA |  |  |  |  |
| ATCC 27090 | *Actinobacillus pleuropneumoniae* | NA | NA | NA | NA |  |  |  |  |
| ATCC 29703 | *Bibersteinia trehalosi* | NA | NA | NA | NA |  |  |  |  |
| ATCC 43625 | *H. somni* | NA | NA | NA | NA |  |  |  |  |
| ATCC 700025 | *H. somni* | NA | NA | NA | NA |  |  |  |  |

^1^Susceptibility to macrolide and tetracycline antibiotics (S = susceptible; R = resistant). MIC breakpoints for macrolide (tulathromycin): $\geq$64 = R; <64 = S. MIC breakpoints for tetracycline: $\geq$8 = R; <8 = S. Tulathromycin represents macrolide antibiotics.

^2^Template cell lysate was prepared using a 1 McFarland Standard of pure culture growth. Ct values for each target are reported using the Rotor-gene Q instrument using threshold values of 0.1. Dynamic tube normalization was used for all analysis. ATCC reference controls were used for specificity evaluation and thus do not have MIC values availability.

**Supplementary materials - Table S5.** Assessment of sufficiency of sample size for determining the optimal cycle threshold value.

| BRD Pathogen | Sample | Class | Antibiotics | No. of Total Samples | No. of Samples with^1^ | | Optimal Cycle Threshold (Ct) | Required Sample Size considering PPV^2^ | | | Required Sample Size considering NPV^2^ | | | Valid^3^ |
| --- | --- | --- | --- | --- | --- | --- | --- | --- | --- | --- | --- | --- | --- | --- |
|  |  |  |  |  | R+I | S |  | Sample Size | Proportion of Cases | Number of Cases | Sample Size | Proportion of Cases | Number of Cases |  |
| *M.haemolytica* | Lung | Tetracycline | Oxytetracycline | 191 | 81 | 110 | 31.00 | 27 | 10% | 3 | 24 | 28% | 7 | YES |
|  |  | Macrolide | Tilmicosin | 191 | 74 | 117 | 33.04 | 27 | 14% | 4 | 17 | 30% | 5 | YES |
|  |  | Macrolide | Tulathromycin | 191 | 67 | 124 | 32.89 | 24 | 13% | 3 | 13 | 34% | 4 | YES |
|  |  | Macrolide | Tilmicosin or tulathromycin | 191 | 75 | 116 | 33.04 | 27 | 14% | 4 | 18 | 30% | 5 | YES |
|  | Nasal | Tetracycline | Oxytetracycline | 72 | 5 | 67 | 32.26 | 17 | 5% | 1 | 12 | 80% | 10 | NO |
|  |  | Macrolide | Tilmicosin | 72 | 2 | 70 | 21.42 | 44 | 1% | 0 | 13 | 50% | 6 | NO |
|  |  | Macrolide | Tulathromycin | 72 | 6 | 66 | 30.73 | 71 | 28% | 20 | 2 | 26% | 1 | NO |
|  |  | Macrolide | Tilmicosin or tulathromycin | 72 | 6 | 66 | 30.73 | 71 | 28% | 20 | 2 | 26% | 1 | NO |
| *P. multocida* | Lung | Tetracycline | Oxytetracycline | 96 | 30 | 66 | 36.10 | 23 | 15% | 3 | 10 | 43% | 4 | YES |
|  |  | Macrolide | Tilmicosin | 95 | 16 | 79 | 32.91 | 44 | 23% | 10 | 3 | 25% | 1 | YES |
|  |  | Macrolide | Tulathromycin | 96 | 10 | 86 | 32.91 | 25 | 15% | 4 | 3 | 38% | 1 | YES |
|  |  | Macrolide | Tilmicosin or tulathromycin | 95 | 16 | 79 | 32.91 | 44 | 23% | 10 | 3 | 25% | 1 | YES |
|  | Nasal | Tetracycline | Oxytetracycline | 78 | 15 | 63 | 29.35 | 32 | 17% | 6 | 4 | 28% | 1 | YES |
|  |  | Macrolide | Tilmicosin | 80 | 6 | 74 | 36.40 | 168 | 36% | 60 | 2 | 41% | 1 | NO |
|  |  | Macrolide | Tulathromycin | 78 | 4 | 74 | 31.47 | 157 | 34% | 54 | 1 | 29% | 0 | NO |
|  |  | Macrolide | Tilmicosin or tulathromycin | 80 | 7 | 73 | 32.22 | 288 | 39% | 112 | 2 | 27% | 1 | NO |
| *H. somni* | Lung | Tetracycline | Oxytetracycline | 93 | 49 | 44 | 36.28 | 32 | 23% | 7 | 62 | 48% | 30 | YES |
|  |  | Macrolide | Tilmicosin | 93 | 21 | 72 | 33.08 | 56 | 29% | 16 | 5 | 34% | 2 | YES |
|  |  | Macrolide | Tulathromycin | 93 | 28 | 65 | 31.67 | 40 | 23% | 9 | 8 | 29% | 2 | YES |
|  |  | Macrolide | Tilmicosin or tulathromycin | 93 | 32 | 61 | 33.08 | 39 | 23% | 9 | 11 | 30% | 3 | YES |
|  | Nasal | Tetracycline | Oxytetracycline | 29 | 13 | 16 | 32.85 | 32 | 20% | 6 | 30 | 63% | 19 | NO |
|  |  | Macrolide | Tilmicosin | 29 | 3 | 26 | 30.88 | 112 | 34% | 38 | 2 | 40% | 1 | NO |
|  |  | Macrolide | Tulathromycin | 28 | 7 | 21 | 27.19 | 36 | 11% | 4 | 31 | 83% | 26 | NO |
|  |  | Macrolide | Tilmicosin or tulathromycin | 28 | 9 | 19 | 26.83 | 36 | 12% | 4 | 40 | 83% | 33 | NO |
| At least one BRD pathogen | Lung | Tetracycline | Oxytetracycline | 296 | 132 | 164 | 36.06 | 25 | 18% | 4 | 23 | 44% | 10 | YES |
|  |  | Macrolide | Tilmicosin | 295 | 101 | 194 | 33.08 | 30 | 18% | 6 | 11 | 31% | 3 | YES |
|  |  | Macrolide | Tulathromycin | 296 | 95 | 201 | 32.89 | 28 | 17% | 5 | 10 | 32% | 3 | YES |
|  |  | Macrolide | Tilmicosin or tulathromycin | 295 | 109 | 186 | 33.08 | 30 | 17% | 5 | 14 | 29% | 4 | YES |
|  | Nasal | Tetracycline | Oxytetracycline | 108 | 25 | 83 | 32.81 | 22 | 16% | 3 | 7 | 52% | 4 | YES |
|  |  | Macrolide | Tilmicosin | 108 | 11 | 97 | 31.82 | 48 | 26% | 12 | 2 | 33% | 1 | NO |
|  |  | Macrolide | Tulathromycin | 108 | 16 | 92 | 31.47 | 110 | 33% | 37 | 2 | 25% | 0 | NO |
|  |  | Macrolide | Tilmicosin or tulathromycin | 108 | 21 | 87 | 31.82 | 123 | 35% | 42 | 3 | 25% | 1 | NO |

^1^S, R, I denotes to susceptible, resistant, and intermediate resistant to the drug classified based on MIC test and CLSI breakpoints, and the categories of R and I are combined into a new category of “resistant”, or R+I. ^2^Minimum required sample size and the proportion of resistant samples (R+I) calculated considering PPV and NPV of importance, respectively. ^3^Valid takes value “YES” if the total sample size and number of resistant samples are both sufficient when considering both PPV and NPV of importance to calculate the optimal threshold based on sensitivity, specificity, and prevalence.

**Supplementary materials - Table S6.** Comparison of 5-fold cross-validation with the optimal cycle threshold (Ct) cutoff value obtained using ROC curves on the overall data.

| Sample | Class | Antibiotics | No. of Samples with^1^ | | Overall Data | | 5-Fold Cross Validation | | |
| --- | --- | --- | --- | --- | --- | --- | --- | --- | --- |
|  |  |  |  |  | Optimal Cycle Threshold (Ct) | Kappa (κ) | Average Optimal Cycle Threshold (Ct) (95% CI) | Kappa (κ) on Train Data –  Average (Standard Deviation) | Kappa (κ) on Test Data –  Average (Standard Deviation) |
|  |  |  | R+I | S |  |  |  |  |  |
| Lung | Tetracycline | Oxytetracycline | 132 | 164 | 36.06 | 0.64 | 35.66  (34.8 – 36.5) | 0.64 (0.04) | 0.61 (0.17) |
|  | Macrolide | Tilmicosin | 101 | 194 | 33.08 | 0.61 | 33.12  (33.0 – 33.2) | 0.61 (0.04) | 0.60 (0.14) |
|  | Macrolide | Tulathromycin | 95 | 201 | 32.89 | 0.64 | 32.64  (32.2 – 33.1) | 0.65 (0.03) | 0.62 (0.12) |
|  | Macrolide | Tilmicosin or tulathromycin | 109 | 186 | 33.08 | 0.62 | 33.12  (33.0 – 33.2) | 0.61 (0.02) | 0.61 (0.09) |
| Nasal | Tetracycline | Oxytetracycline | 25 | 83 | 32.81 | 0.56 | 33.27  (32.4 – 34.2) | 0.56 (0.04) | 0.46 (0.19) |
|  | Macrolide | Tilmicosin | 11 | 97 | 31.82 | 0.30 | 31.83  (31.6 – 32.1) | 0.31 (0.04) | 0.26 (0.16) |
|  | Macrolide | Tulathromycin | 16 | 92 | 31.47 | 0.24 | 36.10  (32.4 – 39.8) | 0.10 (0.18) | 0.01 (0.09) |
|  | Macrolide | Tilmicosin or tulathromycin | 21 | 87 | 31.82 | 0.24 | 33.30  (30.4 – 36.2) | 0.20 (0.13) | 0.09 (0.12) |

^1^S, R, I denotes to susceptible, resistant, and intermediate resistant to the drug classified based on MIC test and CLSI breakpoints, and the categories of R and I are combined into a new category of “resistant”, or R+I.
