# Supplementary material for: Development of a Multiplex Real-Time PCR Assay for Predicting Macrolide and Tetracycline Resistance Associated with Bacterial Pathogens of Bovine Respiratory Disease
Source: Pathogens. 2021 Jan 13;10(1):64. doi: 10.3390/pathogens10010064 (PMC7828349; doi:10.3390/pathogens10010064)
Supplement: Supplementary file 1 [file pathogens-10-00064-s001.zip › Supplementary materials - Text_R1.docx]

**Supplementary materials - Text**

**Elaboration of Accuracy Evaluation Techniques Used in the Present Study**

To develop a multiplex qPCR assay with relative high diagnostic accuracy for classifying antimicrobial resistance status in clinical specimens, it is contingent on a balance between the samples with positive and negative responses to the outcomes of interest, i.e., “resistant” and susceptible [1]. In order to evaluate if the balance is reached, the minimum required sample size along with the required proportion of resistant samples was estimated and compared with the sample sizes and resistant samples included in this study to validate the optimal Ct cutoff values determined using the ROC approach.

PPV and NPV as introduced in section 4.6.1 are considered important factors for estimating the required sample size for evaluating diagnostic accuracy, which denotes the total samples including those are resistant and susceptible. The minimum required number of samples size considering PPV and NPV of importance assuming that the prevalence of the disease (for this study - number of cases R+I) denoted by $\omega$ should neither be too small nor too large ($\omega\neq0 or 1)$ are shown in Equations (1) and (2) respectively and are denoted by $N_{PPV}$ and $N_{NPV}$ :

| $N_{PPV}=\frac{\left( Z_{1-\alpha}+Z_{1-\beta} \right)^{2}*\sigma_{1}^{2}}{\left\{ \phi_{1}-\log\left( \frac{\omega*\gamma}{\left( 1-\omega\right)*\left( 1-\gamma\right)} \right) \right\}^{2}}$ | 1 |
| --- | --- |
| $N_{NPV}=\frac{\left( Z_{1-\alpha}+Z_{1-\beta} \right)^{2}*\sigma_{2}^{2}}{\left\{ \phi_{2}-\log\left( \frac{(1-\omega)*\gamma}{\omega*\left( 1-\gamma\right)} \right) \right\}^{2}}$ | 2 |

$\phi_{1}=log\frac{1-Sp}{Se}$, and $\phi_{2}=log\frac{1-Se}{Sp}$

$\sigma_{1}^{2}=\frac{1-Se}{Se*\omega}+\frac{Sp}{\left( 1-Sp \right)*(1-\omega)}$ and $\sigma_{2}^{2}=\frac{Se}{(1-Se)*\omega}+\frac{1-Sp}{Sp*(1-\omega)}$

where $\alpha=0.05$ is the significance level and $\beta=0.80$ is the power, $Z_{1-\alpha}$ and $Z_{1-\beta}$ are the standard normal values for the corresponding values of $\alpha$ and$\beta$, $\gamma<1-\omega$, $\phi_{1}$ and $\phi_{2}$ are the diagnostic likelihood log ratios for PPV and NPV respectively, $\sigma_{1}^{2}$ and $\sigma_{2}^{2}$ denotes the variances of PPV and NPV and the calculation of $Se$ and $Sp$ can be found in Table 8. Using Equations (1) and (2) the required minimum sample size ($N$) is then calculated by taking the maximum of $N_{PPV}$ and $N_{NPV}$ as shown in Equation 3:

| $N=max(N_{\mathrm{PPV}},N_{\mathrm{NPV}})$ | 3 |
| --- | --- |

The required proportion of resistant samples ($p^{*}$) can then be calculated as shown in Equation (4) with the assumption that $Se+Sp>1 or 100\%$,

| $p^{*}=\left\{ \begin{aligned} \frac{\sqrt{r_{1}}}{1+\sqrt{r_{1}}} , if N_{\mathrm{PPV}}>N_{\mathrm{NPV}} \\ \frac{\sqrt{r_{2}}}{1+\sqrt{r_{2}}} , if N_{\mathrm{PPV}}<N_{\mathrm{NPV}} \\ \frac{\sqrt{r_{1}}}{1+\sqrt{r_{1}}}\mathrm{or}\frac{\sqrt{r_{2}}}{1+\sqrt{r_{2}}}, if N_{\mathrm{PPV}}=N_{\mathrm{NPV}} \end{aligned} \right.$ | 4 |
| --- | --- |

where $r_{1}=\frac{(1-Se)(1-Sp)}{Se Sp}$ , $r_{2}=\frac{SeSp}{(1-Se)(1- Sp)}$.

where $Se$ and $Sp$ are as shown in Table 8. Minimum required number of resistant samples denoted by $N_{p}^{*}$ can then be obtained as shown in Equation (5), where N can be calculated by calculated by Equation (2) or (3).

| $N_{p}^{*}= p^{*}*N$ | 5 |
| --- | --- |

In this study, to evaluate whether the optimal Ct value determined using the ROC approach is considered valid if the observed sample size and the proportion of cases R is greater than or equal to the minimum required sample size $N$ and number of cases $N_{p}^{*}$.

Once the sufficiency of the sample size and required number of resistant samples were determined for detecting the AMR in BRD pathogens, cross-validation technique was further used to evaluate the computational approach used in ROC approach to determine the optimal Ct value. Cross-validation is one of the most common resampling methods to evaluate the performance of a diagnostic test [2]. Herein K-fold cross-validation (CV) technique was used to validate the optimal Ct value of the multiplex qPCR assay for classifying antimicrobial resistance equivalent to the gold standard. In K-fold cross-validation [3], the dataset is split into K equal-sized subsets, the ROC analysis is conducted on K-1 training sets and the misclassification error is calculated on the remaining set. This process is repeated for K times to have an average estimate of the error. Cohen’s $\kappa$ was used to compare the level of agreement between different iteration of K-fold cross-validation. This technique also helped in getting a consistent estimate of the optimal Ct value, which was calculated as an average Ct cutoff value ($AvgCt$) across K iterations, as shown in Equation (6).

| $AvgCt=\frac{1}{K}\sum_{k=1}^{K} Ct_{k}$ | 6 |
| --- | --- |

where K is for K-fold cross validation and $Ct_{k}$ denotes the optimal Ct value obtained in the $k^{th}$ iteration, $k=1,\ldots.,K$

Along with the $AvgCt$, the average $\kappa$ value along with the standard deviation was also calculated for both the training and the test set to compare the level of agreement between qPCR and gold standard test. Equation 7 and 8 shows the average kappa ($\kappa$ ) coefficient for both the training and test set, respectively. The standard deviation of the level of agreement for the training and test set are calculated using equation 9 and 10.

| $Avg\kappa_{tr}=\frac{1}{K}\sum_{k=1}^{K} \kappa_{tr}$ | 7 |
| --- | --- |
| $Avg\kappa_{ts}=\frac{1}{K}\sum_{k=1}^{K} \kappa_{ts}$ | 8 |
| $SD\kappa_{tr}=\frac{1}{K}\sum_{k=1}^{K} {{(\kappa}_{tr}-Avg\kappa_{tr})}^{2}$ | 9 |
| $SD\kappa_{ts}=\frac{1}{K}\sum_{k=1}^{K} {{(\kappa}_{ts}-Avg\kappa_{ts})}^{2}$ | 10 |

where K and $k$ are same as defined after equation 6.

**References**

[1] Steinberg DM, Fine J, Chappell R. Sample size for positive and negative predictive value in diagnostic research using case-control designs. Biostatistics 2009; 10:94-105.

[2] Riley RD, Ahmed I, Debray TPA *et al.* Summarising and validating test accuracy results across multiple studies for use in clinical practice. Stat Med 2015; 34:2081-2103.

[3] T. Hastie RT, and J. Friedman. . The Elements of Statistical Learning. Springer New York Inc.; 2001.
